# Macrophage Polarization Contributes to the Anti-Tumoral Efficacy of Mesoporous Nanovectors Loaded with Albumin-Bound Paclitaxel

**DOI:** 10.3389/fimmu.2017.00693

**Published:** 2017-06-16

**Authors:** Fransisca Leonard, Louis T. Curtis, Matthew James Ware, Taraz Nosrat, Xuewu Liu, Kenji Yokoi, Hermann B. Frieboes, Biana Godin

**Affiliations:** ^1^Department of Nanomedicine, Houston Methodist Research Institute, Houston, TX, United States; ^2^Department of Bioengineering, University of Louisville, Louisville, KY, United States; ^3^Department of Surgery, Baylor College of Medicine, Houston, TX, United States; ^4^James Graham Brown Cancer Center, University of Louisville, Louisville, KY, United States

**Keywords:** macrophage polarization, nanotherapy, breast cancer, computational modeling, tumor microenvironment

## Abstract

Therapies targeted to the immune system, such as immunotherapy, are currently shaping a new, rapidly developing branch of promising cancer treatments, offering the potential to change the prognosis of previously non-responding patients. Macrophages comprise the most abundant population of immune cells in the tumor microenvironment (TME) and can undergo differentiation into functional phenotypes depending on the local tissue environment. Based on these functional phenotypes, tumor-associated macrophages (TAMs) can either aid tumor progression (M2 phenotype) or inhibit it (M1 phenotype). Presence of M2 macrophages and a high ratio of M2/M1 macrophages in the TME are clinically associated with poor prognosis in many types of cancers. Herein, we evaluate the effect of macrophage phenotype on the transport and anti-cancer efficacy of albumin-bound paclitaxel (nAb-PTX) loaded into porous silicon multistage nanovectors (MSV). Studies in a coculture of breast cancer cells (3D-spheroid) with macrophages and *in vivo* models were conducted to evaluate the therapeutic efficacy of MSV-nAb-PTX as a function of macrophage phenotype. Association with MSV increased drug accumulation within the macrophages and the tumor spheroids, shifting the inflammation state of the TME toward the pro-inflammatory, anti-tumorigenic milieu. Additionally, the treatment increased macrophage motility toward cancer cells, promoting the active transport of therapeutic nanovectors into the tumor lesion. Consequently, apoptosis of cancer cells was increased and proliferation decreased in the MSV-nAb-PTX-treated group as compared to controls. The results also confirmed that the tested system shifts the macrophage differentiation toward an M1 phenotype, possessing an anti-proliferative effect toward the breast cancer cells. These factors were further incorporated into a mathematical model to help analyze the synergistic effect of the macrophage polarization state on the efficacy of MSV-nAb-PTX in alleviating hypovascularized tumor lesions. In conclusion, the ability of MSV-nAb-PTX to polarize TAM to the M1 phenotype, causing (1) enhanced penetration of the drug-carrying macrophages to the center of the tumor lesion and (2) increased toxicity to tumor cells may explain the increased anti-cancer efficacy of the system in comparison to nAb-PTX and other controls.

## Introduction

Tumor initiation, growth, and progression rely on the bidirectional interaction of the tumor cells with the cells in the tumor microenvironment (TME). Solid tumors comprise variable amounts of neoplastic and stromal cells. The tumor stroma includes endothelial cells, fibroblasts, and immune cells, mainly macrophages and lymphocytes. Macrophages are a plastic and heterogeneous immune cell population. In particular, tumor-associated macrophages (TAMs), derived from monocytic precursors, comprise the most abundant population of immune cells in the TME (1–3). Macrophages in the TME can undergo functional changes and be polarized from the resting M0 phenotype to the classically activated pro-inflammatory M1 or anti-inflammatory (alternatively activated) M2 general subsets, based on the stimuli in the residing milieu (4). M1 macrophages are characterized by their expression of inducible nitric oxide synthase, production of pro-inflammatory cytokines (e.g., TNF, IL-1, -6, and -12) and reactive oxygen species (ROS). This subpopulation of macrophages promotes strong immune responses and is anti-tumorigenic (5, 6). On the contrary, M2 macrophages antagonize the inflammation and are present in the advanced stages of the healing process. M2 macrophages enhance the formation of tumor stroma by recruiting fibroblasts and activating their differentiation to myofibroblasts, causing the release of pro-angiogenic factors that enable recruitment of endothelial progenitor cells and neo-vasculogenesis and suppression of inflammation through decreased production of ROS and pro-inflammatory cytokines (7, 8). While M2 macrophages possess a significant role in host defense and Th2-mediated activation of the humoral immune response, their presence in the TME promotes tumor development. Presence of M2 macrophages and a high ratio of M2/M1 macrophages in the TME are clinically associated with poor prognosis in many types of cancers (9–12).

It is noteworthy that the tight distinction between M1 and M2 macrophages does not fully describe the continuum of their functions and can be considered as a simplified classification of the two sides of the polarization spectrum (13). TAMs are usually considered M2-like macrophages (14–16), which abandon the M1-related innate and adaptive immune responses capable of destroying malignant cells. Changes in the stimuli of the TME can cause reprogramming of macrophages from an M1 phenotype to an M2-activated state and *vice versa* (17, 18). Macrophage reprogramming has been recently shown to inhibit cancer progression and metastasis (19, 20). Controlling the macrophage polarization state in the TME could provide a novel approach to treating related diseases. Reprogramming M2 macrophages toward the M1 subset is an important focus of recent research, with a number of recent publications demonstrating the ability of some nanomaterials to induce macrophages between polarization states (21–23).

Our previous studies have shown that TAMs play a significant role in therapeutic efficacy of albumin-bound paclitaxel (nAb-PTX) loaded into porous silicon multistage nanovectors (MSV) in liver metastasis of breast and lung tumors (24). Although tumor lesions in the liver have inefficient vascularization, we demonstrated an increased concentration of macrophages acting as chemotherapeutic depots near these lesions. This significantly enhanced efficacy and extended survival in two tested animal models of liver metastases. Furthermore, we have mathematically modeled the efficacy of MSV-nAb-PTX nanovectors in 3D tumor models to project MSV-nAb-PTX efficacy in hypovascularized lesions and concluded that the proposed 3D coculture of macrophages and tumor cells serve as a good model for the *in vivo* condition (25). However, based on the integrated experimental and mathematical analysis of the data, it appears that the efficacy of MSV-nAb-PTX was more than expected solely from macrophages acting as a depot for the drug.

Herein, we aim to evaluate the effect of macrophage phenotype on the anti-cancer efficacy of MSV-nAb-PTX, as well as the effect of these nanovectors on macrophage polarization state. For this purpose, the experiments were performed *in vitro* using a validated coculture of breast cancer tumor cells (3D spheroids) with macrophages and *in vivo* in the breast cancer tumor metastasis mouse model. Our *in vitro* and *in vivo* findings show that treatment with MSV-nAb-PTX affected the macrophages to polarize from the M2-type to the anti-tumorigenic M1 phenotype. Additionally, the treatment increased macrophage motility toward cancer cells, promoting the penetration of therapeutic nanovectors into the tumor lesion. These findings were further incorporated into a mathematical model to help analyze the synergistic effect of macrophage polarization state on the efficacy of MSV-nAb-PTX in treating hypovascularized tumor lesions.

## Results

### Efficacy of Macrophage-Associated MSV-nAb-PTX in 3D TME *In Vitro* Model of Hypovascularized Breast Tumor Lesions

In this study, we use a validated TME model of hypovascularized breast tumor lesions, which consist of macrophages surrounding 4T1 cell spheroids. Rapamycin was used as a factor shifting polarization of macrophages toward the M1 phenotype (26), a positive control of macrophage differentiation.

**Figure 1:**
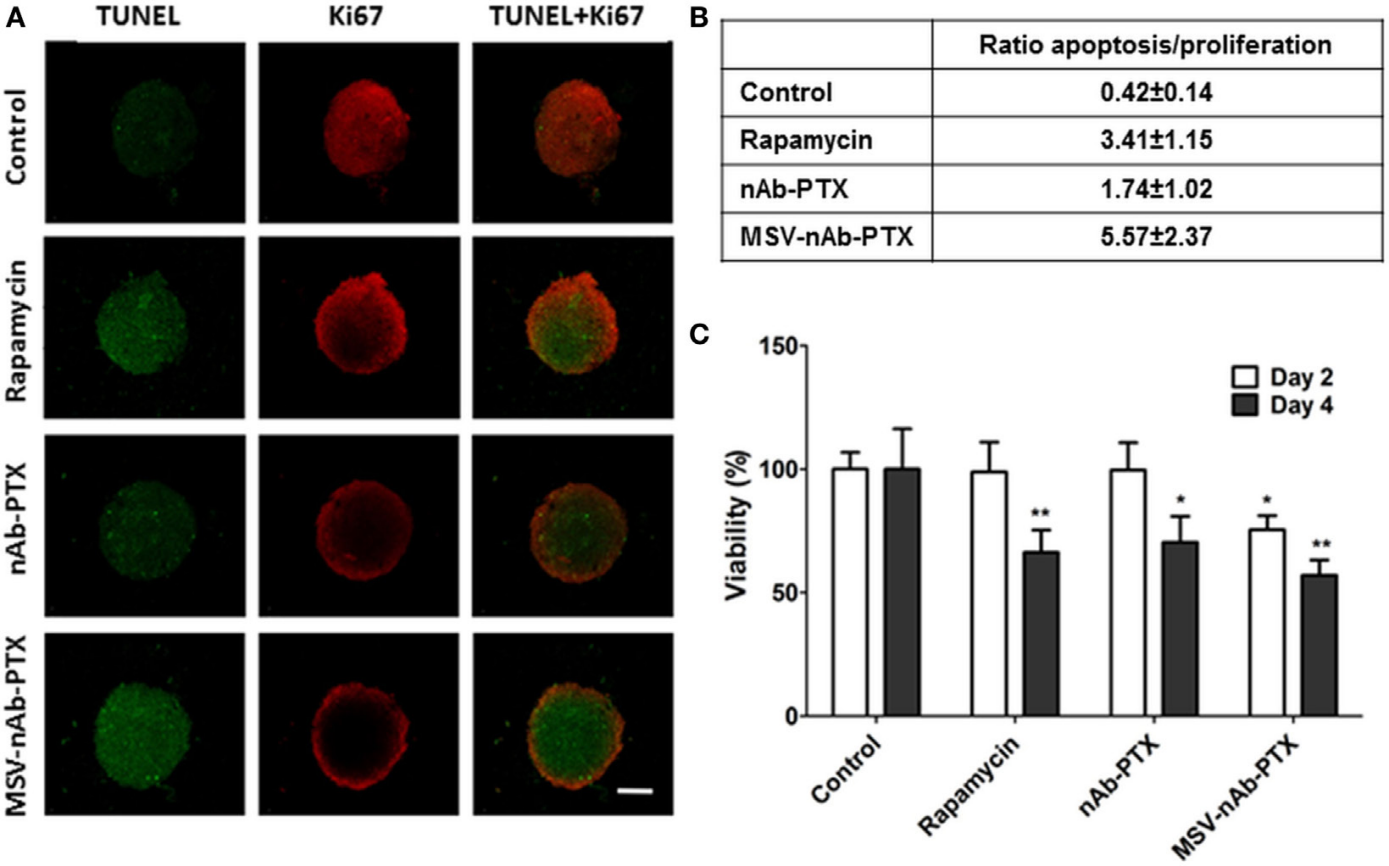
Therapeutic efficacy of the systems in 3D breast tumor tumor microenvironment model. **(A)** Confocal microscopy images from a coculture of 4T1 cancer spheroids and macrophages pretreated with multistage nanovectors (MSV)-albumin-bound paclitaxel (nAB-PTX), nAb-PTX, and rapamycin. Evaluation of apoptosis (terminal deoxynucleotidyl transferase dUTP nick end labeling, green signals) and cell proliferation (Ki67, red signals). **(B)** Ratio of apoptotic/proliferating cell signals as quantified by image analysis (*n* = 6). **(C)** Viability of the breast cancer cells as measured by 3-(4,5-dimethylthiazol-2-yl)-2,5-diphenyltetrazolium bromide assay at 48 and 96 h and normalized to untreated control. Mean ± SD (*n* = 9), *p < 0.05; **p < 0.01 compared to control.

As shown in Figure 1, Ki67 staining indicated that the cells in the control spheres actively proliferated. All treatment groups including nAb-PTX, MSV-nAb-PTX, and rapamycin induced apoptosis in the spheres [terminal deoxynucleotidyl transferase dUTP nick end labeling (TUNEL) staining] and reduced tumor cell proliferation (Figure 1A). Similar to previously reported *in vivo* data (24), treatment with MSV-nAb-PTX and nAb-PTX both resulted in a high apoptosis rate, as shown by green signals from the cells in Figure 1A. Rapamycin induced apoptosis in a similar rate to MSV-nAb-PTX, and cell proliferation was only slightly inhibited by rapamycin. This inhibition was not as efficient as exhibited in the nAb-PTX and MSV-nAb-PTX treatment groups. Spheroids treated with nAb-PTX displayed low proliferation profiles, as observed by a weak Ki67 signal, mostly within the ~75 μm of the outer layer of the spheres. In the MSV-nAb-PTX-treated group, the effect was more pronounced and only cells within ~20 μm from the outer layer of the spheroids were still proliferating (Figure 1A). The ratio of the tumor cells undergoing apoptosis/proliferation is in the following order: MSX-nAb-PTX > rapamycin > nAb-PTX > untreated control (Figure 1B). Furthermore, 3-(4,5-dimethylthiazol-2-yl)-2,5-diphenyltetrazolium bromide (MTT) assay showed that at 2 days from treatment, tumor cell viability was reduced (by >30%) only in the cells treated with MSV-nAb-PTX. At 4 days, more than 30% of tumor cells were not viable following preincubation of macrophages with nAb-PTX and rapamycin, while MSV-nAb-PTX reduced viability by >60% (Figure 1C).

### Macrophage Pretreatment with MSV-nAb-PTX Shifts Their Phenotype toward M1

**Figure 2:**
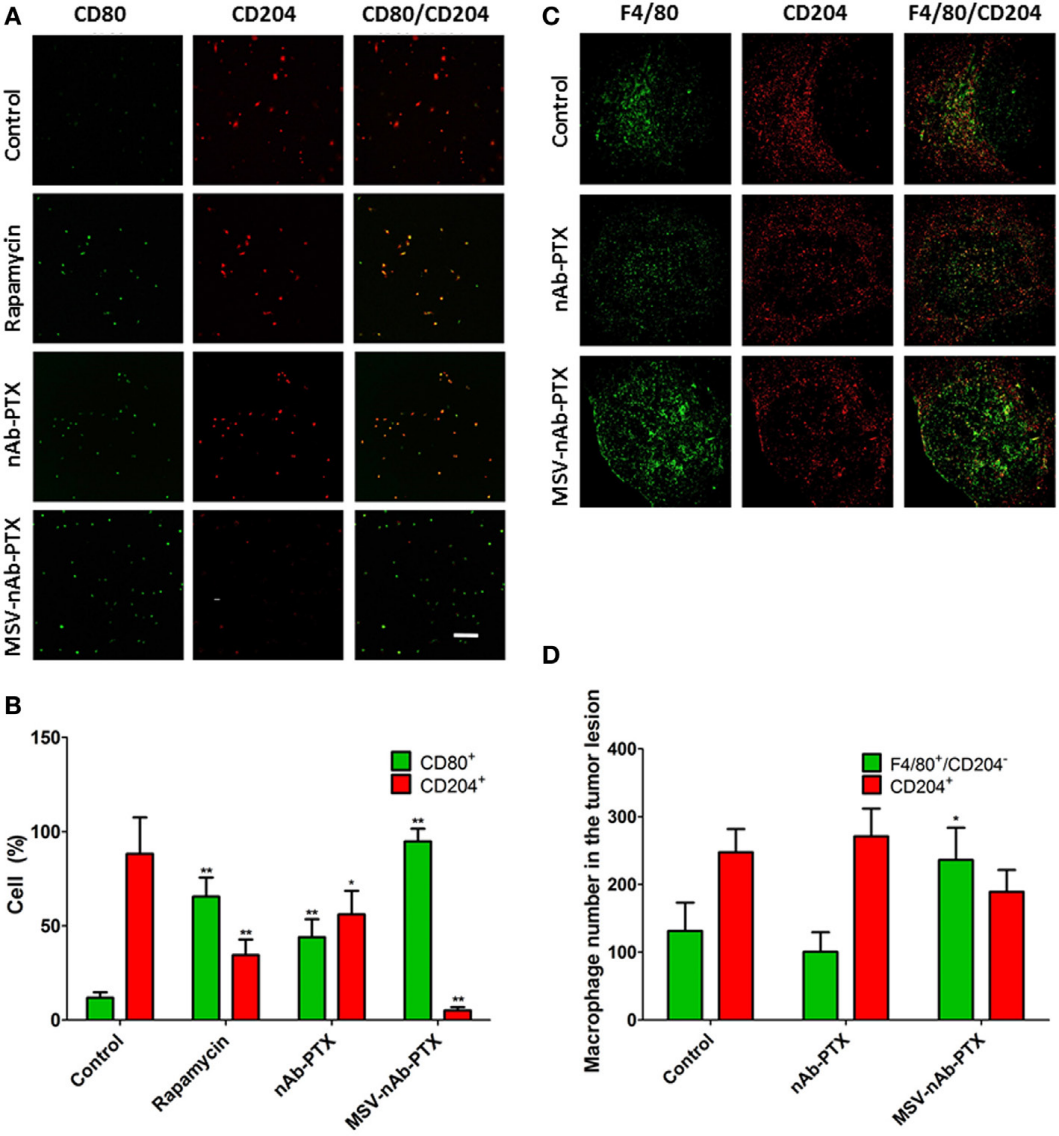
Polarization of macrophages in response to treatment *in vitro* and *in vivo*. **(A)** Confocal microscopy images from a coculture of 4T1 cancer spheroids and macrophages pretreated with multistage nanovectors (MSV)-albumin-bound paclitaxel (nAB-PTX), nAb-PTX, and rapamycin. Macrophages are immunostained for either CD80 (green, M1 marker) or CD204 (red, M2 marker) membranal expression. **(B)**. Quantification of CD80 and CD204 signals from the images presented in **(A)**; **(C)** confocal images of breast tumor lesions in the liver stained for F4/80 (green, total macrophages) and CD204 (red, M2 macrophages). **(D)** Quantification of M1 and M2 signals (M1 number obtained from F4/80^+^/CD204^−^ cells). The results are presented as mean ± SD (*n* = 6–9), *p < 0.05; **p < 0.01 to control.

To investigate the effect of the systems on macrophage polarization state, macrophages pretreated with MSV-nAb-PTX, nAb-PTX, and rapamycin and incubated with breast tumor spheres were tested vs. untreated control for the expression of the cell surface markers CD80 and CD204 (markers for M1 and M2 general polarization states, respectively) (Figure 2). Untreated macrophages in coculture of tumor spheres displayed the M2-like phenotype, as indicated by >85% of the population positive to CD204 staining (Figures 2A,B). This finding is in line with the general polarization of TAM toward the M2 phenotype, as documented previously (5). More than 96% of macrophages in coculture shifted to an M1-like phenotype (CD80 expression) following the treatment with MSV-nAb-PTX. In nAb-PTX- and rapamycin-treated systems, 44.0 ± 9.6 and 65.6 ± 10.1% of cells expressed M1 membrane marker.

We have further confirmed these findings *in vivo* (Figure 2C) in the mouse model of liver metastasis of breast tumors. The predominant population of macrophages in the untreated control group was of M2-like polarization state. MSV-nAb-PTX significantly shifted the population of macrophages toward the M1 phenotype (by twofold), while nAb-PTX had no effect on the macrophage polarization state (Figure 2D). Interestingly, more macrophages were present in the breast cancer metastatic liver lesions treated with MSV-nAb-PTX, which prompted us to look for the effect of nanovectors on macrophage migration.

### Effect of MSV-nAb-PTX on Macrophage Migration toward and into 4T1 Cancer Cell Spheres

**Figure 3:**
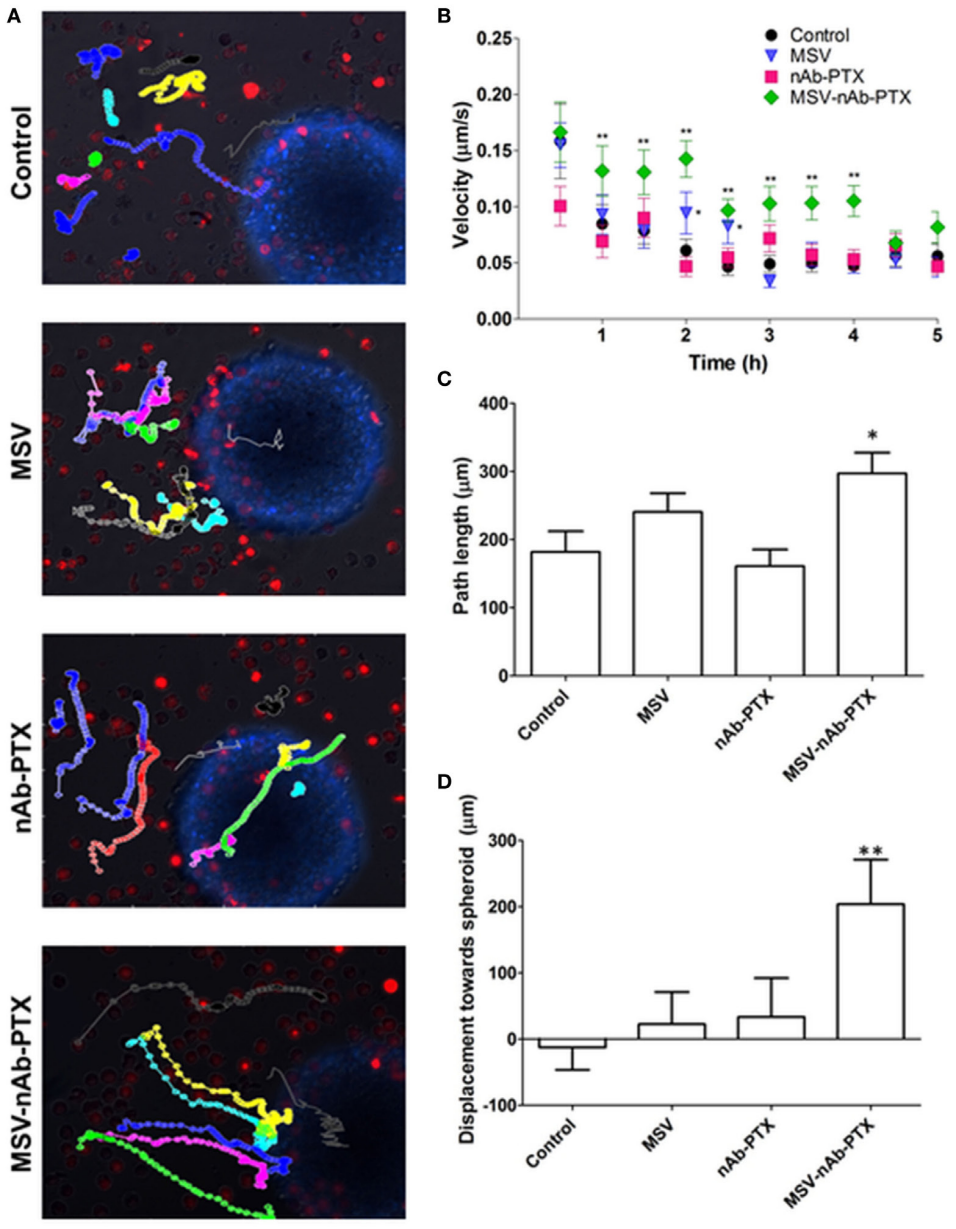
Tracking of macrophage migration kinetics, directionality, and dynamics as a function of treatments. **(A)** Macrophage trajectories following the preincubation with multistage nanovectors (MSV)-albumin-bound paclitaxel (nAb-PTX), nAb-PTX, MSV, and no treatment control were tracked relative to the movement of the tumor spheres. Individual trajectories are presented in different colors. Red: macrophages stained DiD membrane dye. Blue: 4T1 breast tumor cells stained with Hoechst 33342 nucleus dye. The trajectories were tracked by live imaging for 5 h and analyzed for velocity **(B)**, path length **(C)**, and directionality **(D)** based on the displacement toward the breast tumor. The results are presented as mean ± SEM (*n* = 20–25), *p < 0.05; **p < 0.01 as compared to control.

In order to evaluate the effect of MSV-nAb-PTX pretreatment on macrophage migration toward the tumor spheres and into the sphere core, experiments were performed in the 3D TME model we previously developed (25). Time-lapse videos of live-cell images of pretreated macrophages introduced to the tumor spheroids have shown specific directionality and enhanced speed of macrophages pretreated with MSV-nAb-PTX as compared to controls (Figure 3). NIS elements analysis of the videos revealed an increased speed of macrophages treated with MSV-nAb-PTX within the first 5 h (Figure 3B). The increased speed does correlate with a slight increase in path length of the distance traveled by MSV-nAb-PTX macrophages (Figure 3C). All other treatments did not alter the path length compared to the control. However, the most significant change was observed in the directionality of the macrophage migration. The analysis of macrophage displacement toward the tumor sphere within the 5 h time frame showed a significantly specific movement toward the tumor spheres by the macrophages treated with MSV-nAb-PTX. On the other hand, no specific directionality in the movement of macrophages was observed in the cells treated with MSV or nAb-PTX (Figure 3D).

**Figure 4:**
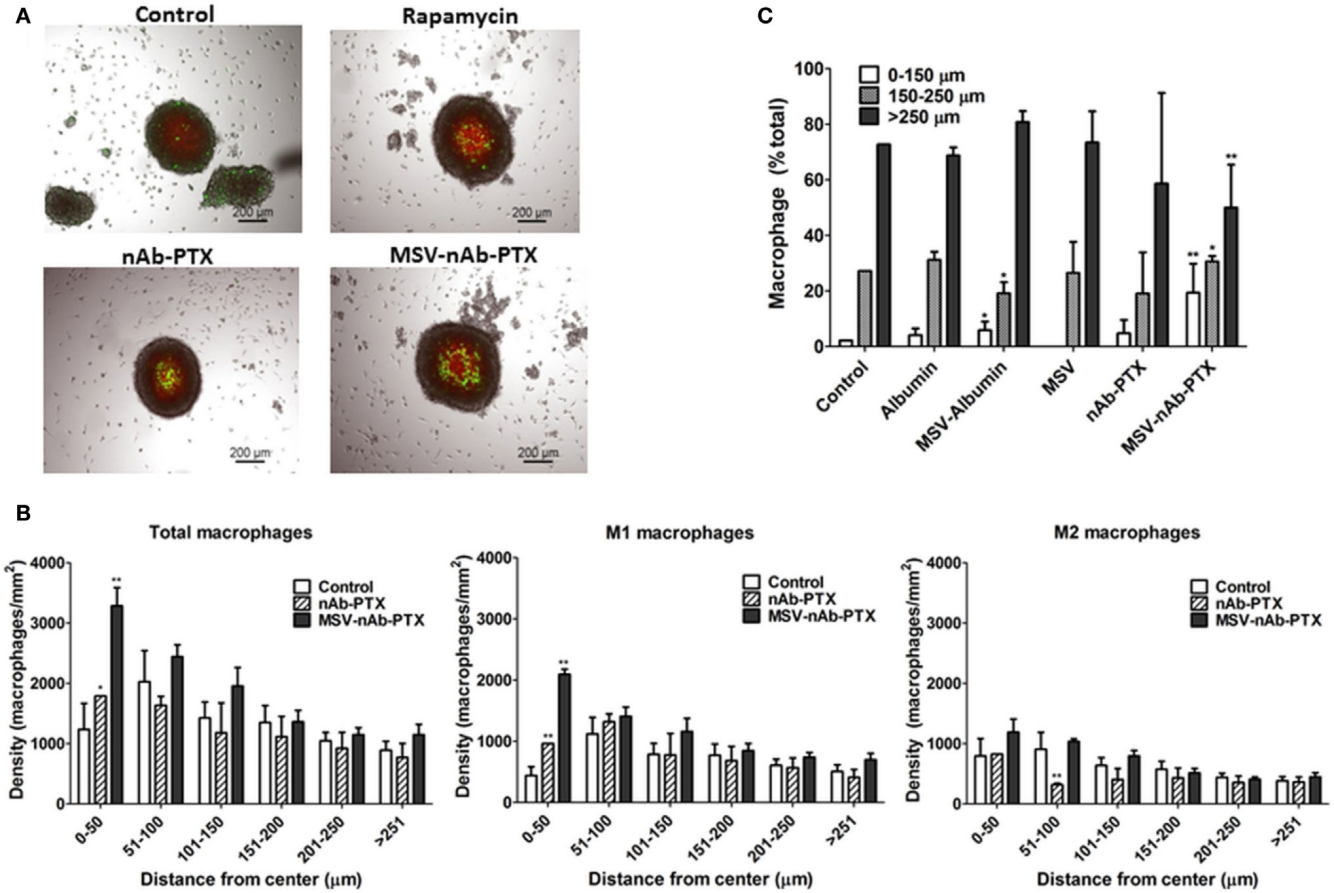
Depth of penetration of macrophages into the breast cancer spheroids. **(A)** Bright-field and fluorescence composite images showing the localization of macrophage (green, DiO membrane dye, Invitrogen) within 4T1 breast cancer cell spheroids (red, DiD membrane dye, Invitrogen) as a function of treatment with multistage nanovectors (MSV)-albumin-bound paclitaxel (nAb-PTX), nAb-PTX vs. untreated control. **(B)** Quantitative analysis of macrophage localization in relation to the center of the sphere. Spheres were segmented into six regions of 50 μm each starting from the center of the tumor sphere (0–50, 51–100, 101–150, 201–250, and >251 μm). Total number of macrophages and numbers of M1 and M2 macrophages were counted within each region and displayed as macrophage density. **(C)** Individual components of MSV-nAb-PTX, namely, albumin, MSV-albumin, MSV, nAb-PTX, and the whole vector, MSV-nAb-PTX, were tested for their ability to increase the number of macrophages inside the deep layers of the tumor spheres. The results are presented as mean ± SD (*n* = 9), *p < 0.05; **p < 0.01 compared to control.

Macrophages pretreated with various systems were tracked and counted in the different depths of the tumor sphere, in increments of 50 µm (Figure 4), focusing on the central part of the spheroid (average diameter 450–500 μm). The density of macrophages in the deep layers of the tumor sphere significantly increased (>2-fold compared to control) after they were pretreated with MSV-nAb-PTX treatment. nAb-PTX only caused moderate increase in the macrophage number in the innermost layer of the spheres. Further analysis revealed that most of the macrophages found in the center of the spheres were M1-like phenotype. These data correlate well with an *in vivo* analysis of macrophage localization in breast cancer liver metastatic lesions previously published (25).

We further tested various components of the MSV-nAb-PTX system to determine the factors crucial for macrophage motility toward the center of the sphere (Figure 4C). Various elements of MSV-nAb-PTX were tested for their effect on macrophage motility: fluorescently labeled albumin (Ab) as a major component of nAb-PTX; MSV; MSV-Ab; and nAb-PTX. MSV did not affect the number of macrophages in the center of the tumor spheroid as compared to untreated control, while Ab, MSV-Ab, and nAb-PTX, surprisingly, slightly increased it. MSV-nAb-PTX enabled an increased migration of the macrophages into the deep layers of the tumor sphere. The number of macrophages in the deep layers of the tumor sphere treated with MSV-nAb-PTX was more than the summary of the effects of all individual components of the system, pointing toward the potential synergy of the factors being involved.

### Effect of MSV-nAb-PTX Pretreatment of Macrophages on Cytokine Production by the Tumors *In Vitro* and *In Vivo*

**Figure 5:**
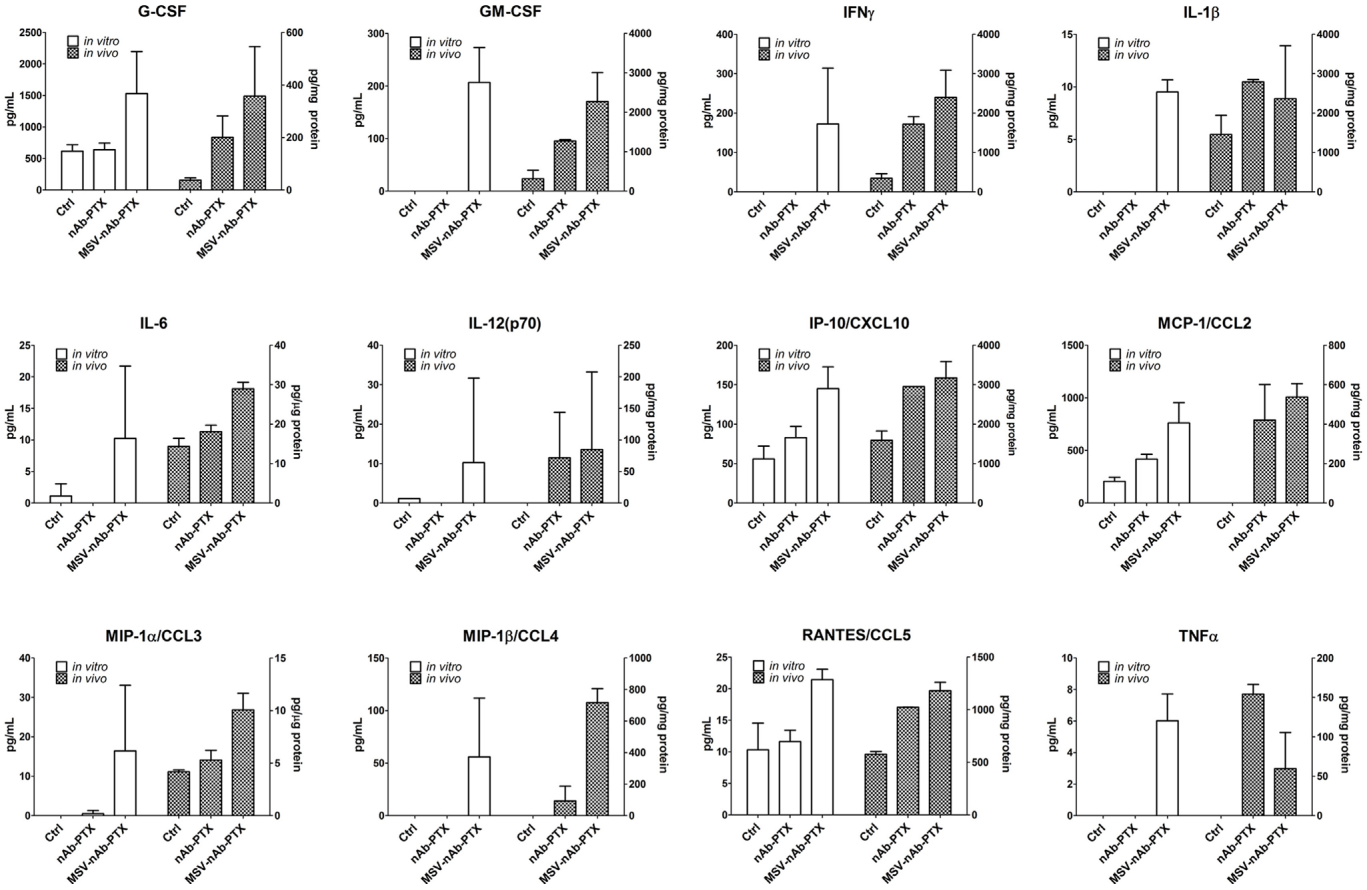
Effect of pretreatment of macrophages with multistage nanovectors (MSV)-albumin-bound paclitaxel (nAb-PTX) vs. nAb-PTX and control of cytokine production by the tumors *in vitro* and *in vivo*. Cytokine release was analyzed using MILLIPLEX MAP Mouse Cytokine/Chemokine Immunology Multiplex Assay (EMD Millipore, Billerica, MA, USA) and measured by Luminex 200™ (Luminex, Austin, TX, USA). For *in vitro* evaluation, the systems were preincubated with macrophages and the conditioned media were introduced to the tumor spheres and incubated for 2 days. For the *in vivo* study, liver metastatic lesions as well as the surrounding area of the lesion (tumor microenvironment) were dissected and processed for analysis as described in Section “Materials and Methods.”

The main function of the macrophages in the TME is tightly related to their interaction with cancer cells, resulting in the secretion of soluble factors that shape the tumor milieu. Therefore, we further performed a thorough analysis of the cytokines and chemokines in the TME 3D model *in vitro* and in hepatic metastases of cancerous breast lesions *in vivo*. Interestingly, neither nAb-PTX nor MSV-nAb-PTX had an effect on the release of the cytokines from the macrophages following direct incubation with the systems (Figure S1 in Supplementary Material). The quantification of the factors released by the tumor cells as a response to the conditioned media from macrophages pretreated with nAb-PTX and MSV-nAb-PTX *in vitro* and *in vivo* is summarized in Figure 5. Cytokine levels measured in tumor cells in response to conditioned media and *in vivo* follow very similar trends. The following factors were increased in both *in vitro* and *in vivo* settings in the TME for MSV-nAb-PTX: G- colony-stimulating factor (CSF), GM-CSF, IFN-gamma, IL-1beta, IL-6, IL-12, IP-10, CCL3, CCL4, CCL5, and TNF-alpha. These data point toward the significant effect of the whole MSV-nAb-PTX system on the macrophage polarization state *in vitro* and *in vivo*. Interestingly, only MCP-1 increased for nAb-PTX-treated macrophages both *in vitro* and *in vivo* settings.

### Mathematical Modeling to Simulate Effect of Macrophage Polarization on Tumor Response *In Vivo*

In order to further analyze the treatment efficacy of MSV-nAb-PTX, we mathematically modeled the effect of MSV-nAb-PTX on hypovascularized liver lesions *in vivo* coupled with macrophage differentiation into M1 and M2 subtypes. As in our previous work (25), the lesion growth was simulated in parallel with the dynamic drug distribution.

**Figure 6:**
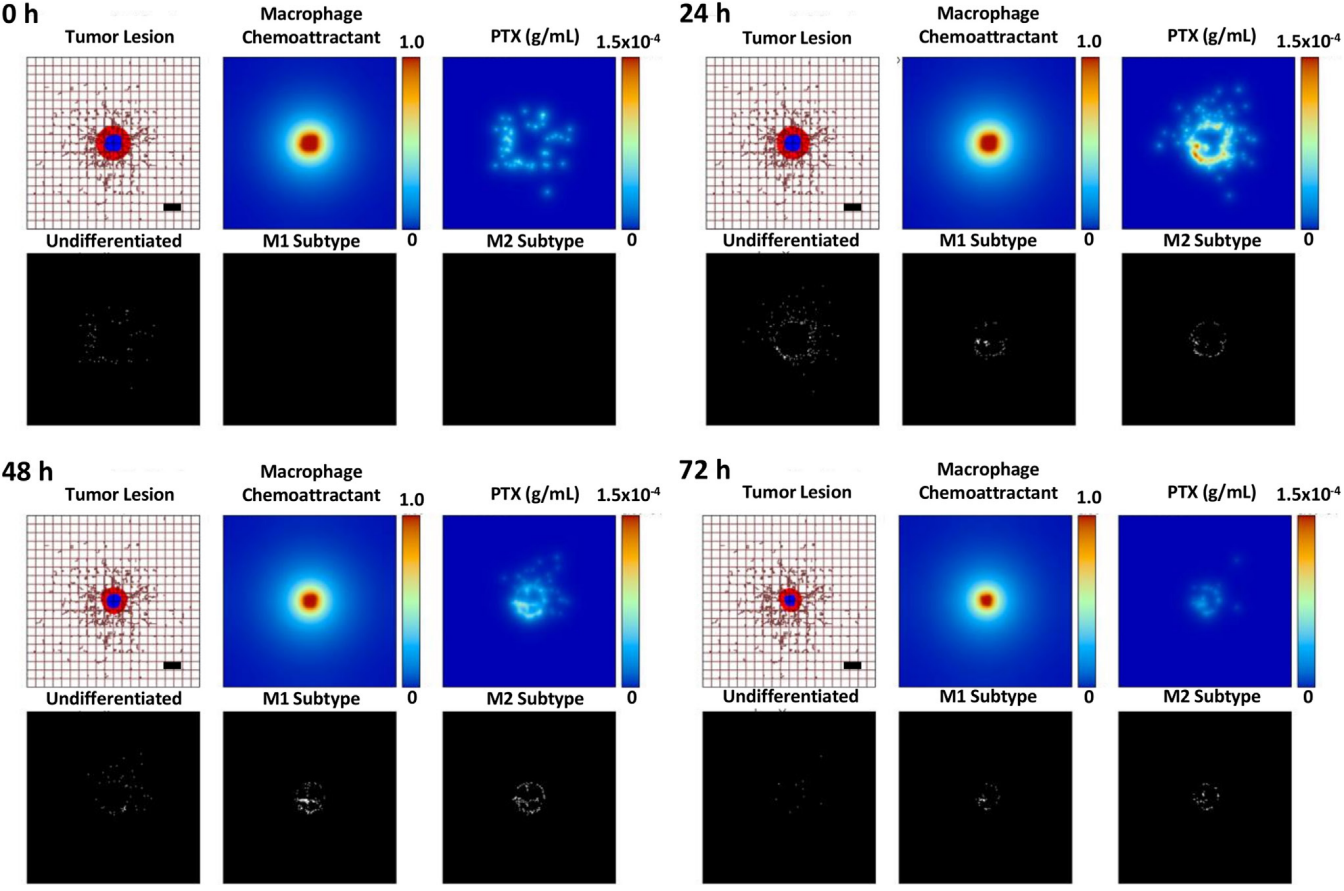
Simulation of breast cancer liver metastasis therapy with multistage nanovectors (MSV)-albumin-bound paclitaxel (nAb-PTX) over the course of 72 h post treatment initiation. For each of the four sets of panels presented for post therapy initiation (0, 24, 48, and 72 h), the tumor lesion is shown (upper left panels) with viable tumor tissue (red) enclosing a hypoxic region (blue) without necrosis. The dense liver capillary network is modeled by the rectangular grid (brown), with irregular sprouts generated through angiogenesis during the lesion progression. Individual macrophages (white dots, lower left panels) are recruited to the vicinity of the lesion based on chemoattractants released by the tumor cells (upper middle panels) and as a response to MSV-nAb-PTX therapy. During this process, the macrophages differentiate into M1 (white dots, lower middle panels) or M2 subtypes (white dots, lower right panels), which, respectively, either hinder or aid the tumor progression. The MSV-nAb-PTX is retained near and within the lesion by the macrophage infiltration, while the drug is slowly released from them in the tumor proximity (upper right panels). The effect of the therapy on the overall lesion size is evident by 72 h. Bar = 200 µm.

Figure 6 illustrates the effects of therapy with the MSV-nAb-PTX-loaded macrophages. Undifferentiated macrophages extravasate from the vasculature and migrate toward the lesion based on the chemotactic gradient of attractants (such as pro-angiogenic factors released by tumor cells) in the surrounding microenvironment. During this process, the macrophages differentiate into M1 or M2 subtypes depending on the ratio of pro-M1 and pro-M2 macrophage factors being released by viable tumor cells in response to the MSV-nAb-PTX system. M1 macrophages are simulated to release nitric oxide, which inhibits cell viability, while M2 macrophages release tumor growth factors, which promote cellular proliferation (5). Each macrophage acts as a source of drug to simulate the release of PTX from the MSV-nAb-PTX formulation. With MSV-nAb-PTX at 24 h post single treatment, the tumor has slightly shrunk (top right) compared to the initial lesion (5% radius decrease), while the drug is being released by the macrophages. At 72 h, the lesion radius attains the highest regression (68% of its original size), by which time most of the drug has been released from the surrounding macrophages. These results are consistent with our previous modeling work (25).

**Figure 7:**
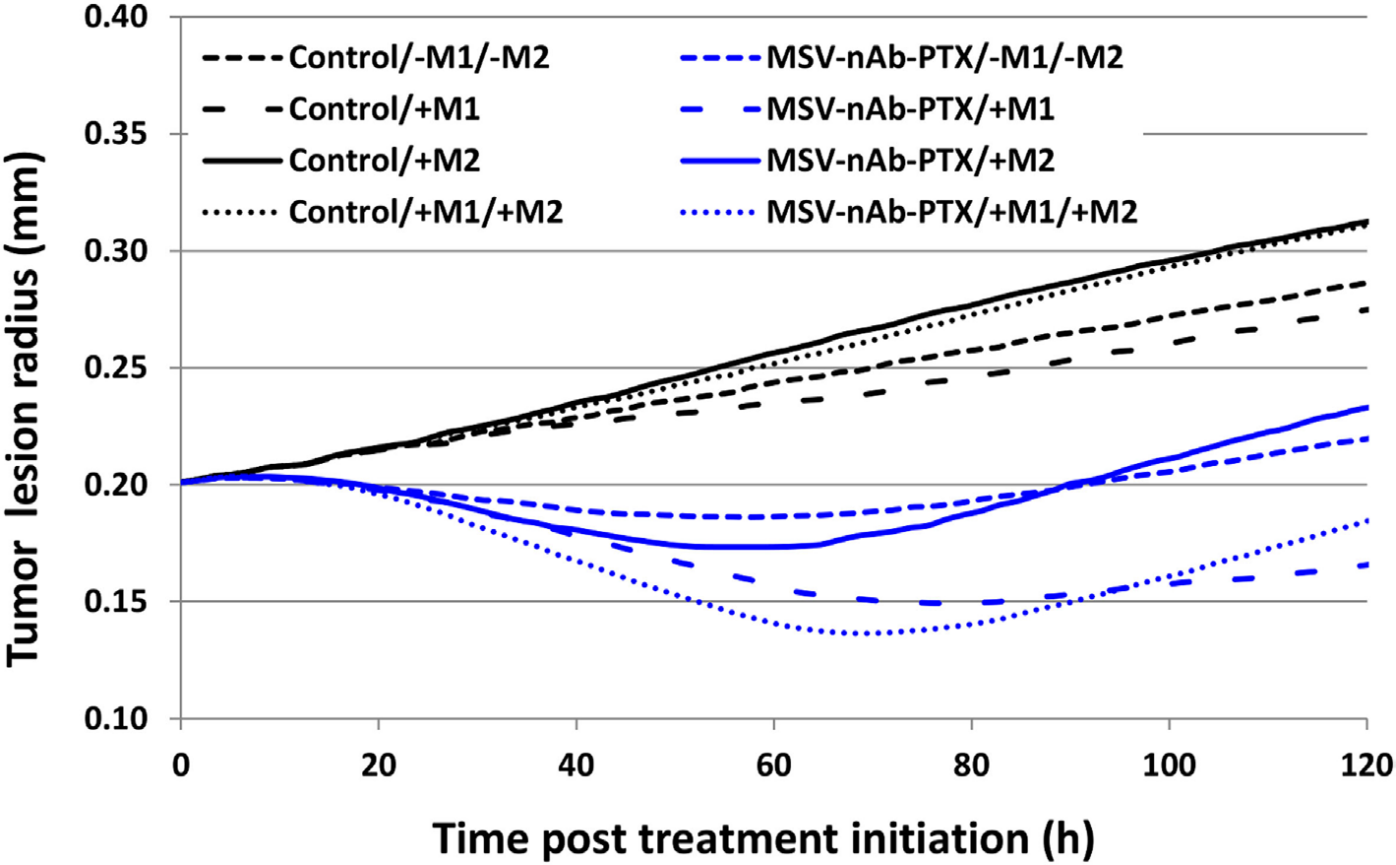
Simulation of tumor progression for untreated and multistage nanovectors (MSV)-albumin-bound paclitaxel (nAb-PTX)-treated cases including various combinations of macrophage polarizations simulated over the course of 5 days post treatment initiation. Control: untreated tumors; MSV-nAb-PTX: tumors treated with the therapeutic system. M1 and M2 refer to the addition of the polarized macrophage populations to the model. The cytotoxic effect of the M1 subtypes is simulated to affect the tissue proportional to the concentration of nitrous oxide released in the immediate vicinity of the macrophage. M2 macrophages release diffusible growth factors which promote tumor proliferation. In addition, treated macrophages (M1 and M2) are simulated to release drug which only affects proliferating tissue due to the cell cycle-dependent effect of PTX.

Figure 7 compares the relative contribution of the macrophage polarization in conjunction with MSV-delivered drug to the tumor progression over the course of 5 days after a single treatment. As expected, the cases without treatment are projected to grow unbounded, with the M2-only and the M1/M2 cases attaining 157 and 156% of their original radius, respectively, while the case without M1/M2 or M1-only, respectively, reaches 143 or 138%. In contrast, all of the MSV-nAb-PTX-treated cases experience regression, which is modulated by the contribution of the macrophage differentiation. The most therapeutically effective scenario is the case with M1-only, reaching 83% of the original radius, followed by the case with both M1 and M2 present, attaining 94%. The cases with M2-only and without any macrophages are anticipated to reach 118 and 111% of their original radius, respectively. Interestingly, the model projects that the presence of the M2 phenotype enhances drug cytotoxicity due to the M2 tumor growth-promoting effect enlarging the subset of the tumor population that is susceptible to the cell cycle-specific activity of PTX. However, over the long term, the cases with M2 macrophages recover faster than the cases without their presence, thus promoting tumor growth.

## Discussion

It is currently well recognized that the fine interplay between deregulation of tumor cells and the cells of the TME is imperative for all stages of tumor development (27). Macrophages represent the major population of infiltrating immune cells in TME (28). Macrophage polarization is detrimental in the development and progression of cancer (28). TAMs generally belong to the subclass of alternatively differentiated, M2-like macrophages. They have been shown to modulate tissue remodeling and angiogenesis, suppress T cell proliferation, and play a significant role in tumor survival (5). High M2 macrophage density has been clinically correlated with poor prognosis in several epithelial cancers, including breast cancer (29) and hepatocellular carcinoma (30).

On the other hand, clinical studies have shown that an increased M1/M2 ratio in the TME is linked to extended survival in ovarian (31), gastric (10), colorectal (32), and lung (33) tumors. M2-like TAMs are characterized by a constitutive high expression of multiple tumor growth promoting factors, including VEGF, FGF1 and 2, PDGF, GM-CSF, insulin-like growth factor-1, and TGF-β (34). For example, in a mouse model of breast cancer, expression of CSF-1 was highest at the invasive edge of the malignancy, which was consequently enriched with M2 macrophages. Epidermal growth factor released by these macrophages increased tumor cell migration and metastasis (35). Flexibility and plasticity represent the key characteristics of the cells of mononuclear phagocytic system and their activation states (5, 36) Polarization of the macrophages between the M1 and M2 general subtypes can be reversed, as was shown in *in vitro* and *in vivo* studies (37). Pathological changes in inflammatory states can sculpture this transition, with M1 macrophages present at initiation and during progression of the inflammatory process and M2 macrophages participating in its resolution. In cancer, histidine-rich glycoprotein (a host-produced protein deposited in the stroma) was shown to induce TAM reprogramming from M2 to M1, resulting in vascular normalization and improved response to chemotherapy (38).

Our previous study also identified the enrichment of macrophages in the tumor periphery of breast cancer liver metastases in a mouse model (24). We have shown that by directing transport of an Ab-bound drug, nAb-PTX, toward the macrophages in the tumor periphery in the liver using MSV, we could increase the concentration of the drug in the lesions and, consequently, the tumor killing efficiency. However, the pronounced anti-tumor effect observed with MSV-nAb-PTX in this study could not be fully explained only by the shift of the concentration of the drug toward the tumor lesions; thus, in the present work we aimed to evaluate the effect of MSV-nAb-PTX on the inflammatory state of the TME, the migratory potential of the macrophages in the tumor lesion and on the interactions of macrophages with the tumor cells. The studies were performed *in vitro* in a previously validated 3D model of hypovascularized breast cancer lesions with macrophages on the lesion periphery (25, 39).

Tumor cell proliferation and apoptosis analysis (Figure 1) confirmed that MSV-nAb-PTX preincubated with macrophages had a pronounced therapeutic efficacy, in line with the *in vivo* data (24). It is important to note that in this experimental set we did not expose the tumor spheres to the drugs directly, but only to the macrophages preincubated with the systems, similar to the *in vivo* situation, where hypovascularized breast cancer lesions in the liver are surrounded by macrophages. Preincubation of macrophages with rapamycin, an mTOR inhibitor that is known to induce the polarization of macrophages toward the M1 phenotype (26), had a mild effect on tumor cell proliferation, but significantly increased the number of apoptotic cells in the lesions; thus showing that M1 polarization induced tumor cell apoptosis.

Furthermore, we have analyzed the number of M1 and M2 polarized macrophages in the tumor lesions and the localization of the macrophages within the tumor cores *in vitro* and *in vivo* (Figures 2–4). As expected, the control (untreated) tumors had increased population of alternatively activated M2-like macrophages. The M2 phenotype is characterized by an improved phagocytic activity (40), since this general subcategory of macrophages fights inflammation and participates in tissue remodeling. M2 macrophages uptake solid particles more efficiently, which helps to concentrate nAb-PTX delivered through MSV. *In vitro*, all treatments shifted this ratio to a new homeostasis, increasing the population of M1 macrophages and decreasing the fraction of M2 macrophages (Figure 2). With MSV-nAb-PTX, this effect was the most prominent, and the population of M1 activated macrophages increased 20-fold while M2 macrophages represented less than 5% of the total number of macrophages. Treatment with nAb-PTX *in vitro* yielded equal populations of M1 and M2 macrophages. Although M1 macrophages predominated with rapamycin, the overall number of macrophages decreased due to a toxic effect of the drug related to inhibition of the mTOR pathway, which is in line with the reported mechanism of rapamycin to induce apoptotic cell death in M0/M2 but not M1 macrophages (26). Interestingly, only MSV-nAb-PTX, but not nAb-PTX caused the shift in the macrophage polarization state *in vivo*. This could be due to the longer retention of the MSV-nAb-PTX in the lesion and specific association of the carrier with the macrophages (24). PTX has been reported to possess a lipopolysaccharide (LPS)-like property, activating murine macrophages by mimicking bacterial LPS through binding to MD2, an extracellular protein of TLR4 (41). LPS-dependent TLR4 can be activated by PTX and internalized into endosomes, activating downstream signaling pathways *via* endocytic shuttling, and therefore promoting polarization of macrophages toward the M1 phenotype. A recent study demonstrated the ability of nAb-PTX to enhance the macrophage activation process due to macropinocytic uptake and the fusing of macropinosomes and endosomes (42). In our study, the increased concentration of PTX in the TME mediated by MSV-nAb-PTX induced the release of pro-inflammatory cytokines by the tumor cells, promoting the pro-inflammatory milieu in the TME and modulating the macrophages to undergo M2 to M1 polarization. Our results also suggest that the Ab component of the nAb-PTX may be involved in this process and slightly increased macrophage migration toward the center of the tumor spheroids (Figure 4), although further study is needed for deeper understanding. Ab has been previously reported to contribute to the intratumoral concentration increase of nAb-PTX *via* binding to the 60 kDa glycoprotein receptor and thus increasing transcytosis (43).

We extended the computational model presented in our previous study by Leonard et al (25) to account for macrophage polarization into M1 and M2 subtypes. The simulations provide a platform to analyze the respective effects of different subsets of macrophages in the tumor in combination with MSV-nAb-PTX therapy with the ultimate goal to optimize treatment outcomes. The modeling results suggest that a single therapy may delay the tumor growth *in vivo* but not completely eradicate the lesion. One reason is that insufficient drug is released by the macrophages in the tumor vicinity to kill all of the tumor cells. Modulation of the macrophage population to increase its size and further drive its polarization toward tumorigenicity, e.g., with an immunotherapy, may achieve a stronger one-time response. However, as shown in our previous study, repeated treatments at regular intervals may still be necessary for complete remission to account for the time it takes for hypoxic (quiescent) cells to resume cycling and thus be sensitive to the chemotherapeutic. We further note that the simulations reflect the variability in experimental measurements regarding the effect of the macrophages. The untreated case with no macrophages and the untreated case with both subtypes could be more similar than shown in Section “Results” (Figure 7), while the effect of the M1 macrophages was calibrated to the low end of possible values. Adjusting for these factors, however, does not affect the overall response difference predicted between untreated and treated cases or the response-modulating effect projected for the M2 macrophages.

Various effects can contribute to the increased efficacy of MSV-nAb-PTX *via* inflammatory modulation. Overall, MSV-nAb-PTX increased the motility and directionality of the macrophages toward the tumor sphere (Figure 3). The increased macrophage recruitment may be a response to the increased chemokine release by tumor cells such as CXCL-10, CCL-2, CCL3, CCL4, and CCL5 (Figure 5). Furthermore, the treatment caused deeper macrophage penetration inside the spheroid/tumor lesions (Figure 4), which can correspond with the apoptotic feedback between the dying tumor cells (Figure 1) and the macrophages bearing MSV-nAb-PTX. It is interesting to note that in the therapeutic concentration tested, macrophage polarization (Figure S1 in Supplementary Material) and viability (24, 25) were not affected by the treatment with any of the tested systems.

In hypovascularized lesions in the liver, tumor cells are not directly exposed to the circulating drugs, while the macrophages present in the liver vasculature are exposed to the intravenously administered drug/particles. To mimic this situation, we further incubated the macrophages with nAb-PTX and MSV-nAb-PTX for 1 h [in clinic, 90% of nAb-PTX is cleared from the circulation in this time frame (44)] and further let the macrophages release soluble factors and the internalized drug, exposing the tumor cells to supernatants from the pretreated macrophages (conditioned media collected at 24 h past drug removal). Cytokine and chemokine profiles were also analyzed in the murine model of liver metastasis. In both the *in vitro* tumor spheres and the *in vivo* murine model, there was a significant increase in the factors associated with M1 macrophage polarization, such as CCR5-binding chemokines (CCL3, CCL4, and CCL5) (45), interleukins (IL-6 and IL-1β), and TNF-α (46). A significant increase in GM-CSF levels released by the tumors *in vitro* and *in vivo* in response to exposure to macrophages preincubated with MSV-nAb-PTX can impart an additional feedback on the M1 polarization state (47), as exposure to GM-CSF was previously shown to promote M1 polarization of the macrophages (47, 48).

These findings could explain the attraction of the macrophages toward the tumor spheres *in vitro* and into the tumor core *in vitro* and *in vivo*. In contrast to previous studies showing that cancer cell apoptosis shifted the phenotype of macrophages toward M2 (49, 50), we have observed that the effects of MSV-nAb-PTX enhanced apoptosis of tumor cells. This can be explained by the fact that there is a direct effect of the system on the macrophage polarization toward the M1 phenotype. Activated M1 macrophages have been recently reported to produce and excrete chitotriosidases (or family 18 chitinases), which can modulate proteases and cause damage to cancer cell membranes (51).

In conclusion, our data demonstrate that macrophages carried MSV-nAb-PTX while not being affected by the therapeutics. The phagocytosis of the solid particles by the macrophages enhanced the drug concentrations inside of these immune system cells (24), consequently enhancing concentrations of the drug released by macrophages in the TME. As a result, tumor cells were exposed to higher concentrations of drug, resulting in enhanced tumor-cell killing, while also inducing an LPS-like effect of PTX as described by Byrd-Leifer et al. (52). This prompted tumor cells to release higher levels of pro-inflammatory cytokines, causing further shift of macrophage polarization toward the anti-tumorigenic M1 phenotype. The data also suggest that polarization of the nanovectors contributes to the toxicity toward cancer cells. Altogether, these phenomena could be utilized to design improved nanovector-based cancer therapies.

## Materials and Methods

### Fabrication of nAb-PTX-MSV

Albumin-bound paclitaxel-multistage nanovectors– or fluorescently labeled Ab loaded, Ab-MSV, were fabricated and characterized as previously described (24, 25). Briefly, MSV with 1 × 0.4 μm (*d* × *h*) dimensions were fabricated in a microelectronics facility *via* photolithography and electrochemical etching and further oxidized with 3-aminopropyl-triethoxysilane (APTES) (24, 53). APTES-MSV were lyophilized using the Freezone Freeze Dry System (Labconco, Kansas City, MO, USA). nAb-PTX (Abraxane^®^, Celgene, Summit, NJ, USA) or Ab (Ab–fluorescein isothiocyanate conjugate, Sigma-Aldrich, St. Louis, MO, USA) concentrated solution was loaded to dried MSV particles in aliquots. Loading was enhanced by a drying process *via* incubation of the particles under low pressure (25, 54). nAb-PTX-MSV was characterized for morphology, zeta potential, and the loading efficiency as described earlier (24, 25).

### Cell Culture

Breast cancer 4T1 cells (ATCC, Manassas, VA, USA) were cultured in Minimum Essential Medium (MEM) with 10% FBS, 1% antibiotic/antimycotic, 1% GlutaMAX, 1% NEAA, 1% MEM vitamin, and 1% sodium pyruvate supplements and maintained in humidified atmosphere at 37°C and 5% CO_2_. Mice macrophages were obtained by isolation from fresh mice bone marrow. Monocytes were washed twice with PBS and erythrocytes were lysed by red cell lysis buffer (Sigma, USA), and cells were filtered with a 70 µm filter (BD Lifesciences, USA). Differentiation of monocytes to resting macrophages was initiated by 7-day incubation with macrophage medium, containing 10% FBS and 1% penicillin/streptomycin in RPMI 1640 medium.

### 3D TME Model: Coculture of Breast Cancer Spheres and Macrophages

Tumor spheres were generated using the Bio-Assembler™ system based on protocols we recently reported (39, 55) and grown to ~450–500 μm diameter before cytotoxicity and migration studies. Depending on the studies, macrophages were treated with rapamycin, Ab, MSV-Ab, nAb-PTX, MSV, or MSV-nAb-PTX for 4 h and stained with Vybrant Cell-Labeling Solutions (Molecular Probes, Eugene, OR, USA). After treatment, supernatants were removed and cells were washed with fresh medium to ensure that no 4T1 cancer cells are not in contact with drugs in the solution (similar to the clinically relevant situation in hypovascularized tumor lesions). Primary macrophages (1 × 10^3^) were cultured together with 4T1 spheres in a 96-well plate and kept in an incubator. Images were taken by fluorescent microscopy after 24, 48, 72, and 96 h and analyzed with NIS-Elements software.

Transwell plates (Corning Inc., Corning, NY, USA) were utilized for coculture study to analyze macrophage differentiation. 4T1 cells (1.5 × 10^4^) were seeded on the apical side while (1.5 × 10^3^) macrophages were seeded on the basolateral side. After 96 h, macrophages were harvested and stained for CD80 and CD204.

### Cell Staining and Confocal Microscopy

Cocultures of macrophages and 4T1 spheres were fixed with 4% paraformaldehyde before staining. Spheres were stained for TUNEL with FITC for apoptosis detection with Promega TUNEL kit (Promega, Madison, WI, USA) according to the manufacturer’s protocol. To assess proliferation the samples were incubated overnight with primary rabbit-anti-mouse Ki67 antibody (1:500, Abcam, Cambridge, UK), washed twice with PBS and incubated with Alexa Fluor 647-labeled goat anti-rabbit secondary antibody for 4 h. Samples were washed twice with PBS before further analysis by confocal microscopy.

Rat anti-mouse CD80 (Thermo Scientific, Rockford, IL, USA) and Alexa Fluor 647-labeled CD204 (Abcam, Cambridge, UK) were utilized for surface marker staining of the macrophages in the coculture. After paraformaldehyde fixation, samples were washed twice with PBS and incubated with 1% BSA for 20 min. CD80 antibody (5 µg/ml) was added and incubated with the samples overnight at 4°C. After washing with PBS, goat anti-rat FITC-labeled antibody was added to the samples for 2 h at RT. Furthermore, samples were washed and stained with Alexa Fluor 647-labeled anti-CD204 (2 h, RT). Prior to confocal microscopy analysis, the samples were washed twice with PBS.

Tumor spheres and macrophages were visualized using a Nikon A1 confocal microscope (Nikon Inc., Melville, NY, USA) based on the fluorescence of the respective probes and analyzed with NIS elements software (Nikon Inc.). Macrophage signal intensity, quantification of macrophages of various phenotypes, and macrophage penetration into the tumor lesion were assessed as below.

### Tracking of Macrophage Migration Kinetics, Directionality, and Dynamics As a Function of Treatments

For the tracking of macrophage migration toward the tumor spheres, the cells were stained with DiD membrane dye (Invitrogen, USA), pretreated with nAb-PTX, MSV, or MSV-nAb-PTX for 1 h and washed. Furthermore, the macrophages were cocultured with breast cancer spheres as described earlier. To differentiate between the two cell populations, 4T1 breast cancer cells were prestained with 1 µg/mL Hoechst 33342 dye (Thermo Scientific). Tumor spheres and macrophage movements were tracked using a live-imaging system Nikon TiEclipse fluorescence microscope (Nikon Inc., USA) over the time course of 10 h and analyzed with NIS elements. The motility of 4T1 spheres was recorded over time and used as the reference for macrophage displacement calculation. Macrophages speed, path length, as well as coordinates were tracked using NIS elements and calculated for their directionality toward 4T1 spheres using the initial coordinates of the cell vs. the tumor sphere as a reference point.

### MTT Assay

3-(4,5-Dimethylthiazol-2-yl)-2,5-diphenyltetrazolium bromide (Sigma, USA) assay was performed to access cell viability. 4T1 spheres in coculture with macrophages were seeded on 96-well plates before treatment. After 48 or 96 h of incubation, the cells were washed twice with PBS and the MTT assay was run based on the manufacturer instructions. The absorbance was determined using a spectrophotometer (Biotek, Winooski, VT, USA) at 570 nm.

### *In Vivo* Model of Breast Cancer Liver Metastasis

Animal studies were performed in accordance with approved protocols by Houston Methodist Research Institute Institutional Animal Care and Use Committee (AUP-0514-0032). Balb/c mice were purchased from Charles Rivers Laboratories and mouse breast cancer liver metastases xenograft were generated by splenic injection of 10^5^ 4T1 tumor cells/100 μL PBS as we previously described (24, 43). Splenectomy was conducted immediately after injection to prevent primary tumor growth in the spleen, and the xenografts were grown for at least 10 days before therapy.

### *In Vivo* Evaluation of Macrophage Quantity within the Microenvironment

For analysis of TME changes in response to therapy *in vivo*, mice with cancer liver metastasis were randomly divided into three groups (*n* = 4): control, nAb-PTX, MSV-nAb-PTX⋅nAb-PTX, and MSV-nAb-PTX containing 75 mg/kg nAb-PTX (7.5 mg/kg PTX) were injected *via* the tail vein. The treatment was repeated every 3 days and the mice were sacrificed after three treatments. The liver was dissected, embedded in OCT compound (Sakura^®^ Finetek USA, Inc., Torrance, CA, USA), and cut in 4 µm sections for histological and immunofluorescence analyses. The frozen sections were fixed with ice-cold acetone and stained with Alexa Fluor 488-tagged rat anti-mouse F4/80 antibody and TRITC anti-mouse CD204 antibody to detect total macrophages and the M2 subpopulation, respectively. We have used the CD204 marker to characterize alternatively polarized M2 macrophages (56, 57) and F4/80 as a marker for general population of macrophages. Cell nuclei were stained with 4,6-diamidino-2-phenylindole, dihydrochloride.

### Cytokines Analysis

For *in vitro* cytokine and chemokine analysis, macrophages were plated in a 96-well plate, with a density of 10,000 cells/well and treated with 150 ng nAb-PTX or MSV-nAb-PTX for 1 h. Drug treatment was removed, cells were washed twice with PBS, and fresh medium was added to the macrophages. This was performed to mimic the clinically relevant situation, as clinical studies with nAb-PTX revealed that more than 90% of the drug is cleared from circulation within 1 h following intravenous administration (44). Supernatants (conditioned media) were harvested from macrophages after 24 h and 50 µL of this conditioned media were added to 50 µL fresh media to culture preformed tumor spheres. Supernatants from 4T1 spheres were harvested after 2 days, and the cytokine and chemokine release was analyzed by MILLIPLEX MAP Mouse Cytokine/Chemokine Immunology Multiplex Assay (EMD Millipore, Billerica, MA, USA) and measured by Luminex 200™ (Luminex, Austin, TX, USA). Additionally, to determine the effect of treatments to the macrophages themselves, the treated macrophages were further cultured with 100 µL fresh medium for 3 days. After incubation, the culture media were collected for a cyto-/chemokine release study.

From the *in vivo* studies, liver metastatic lesions as well as the surrounding area of the lesion (TME) were dissected. Tissues were weighed, 500 µL PBS with 1× HALTTM protease inhibitor cocktail (Thermo Fisher Scientific, Waltham, MA, USA) was added to the samples, and the samples were homogenized using Polytron PT2100 homogenizer (Kinematica AG, Lucerne, Switzerland). Tissue lysates were incubated under constant agitation for 2 h and the supernatants were separated by centrifugation at 10,000 × *g* for 20 min at 4°C. Supernatants containing protein extracts were used for cyto-/chemokine measurements. Protein content of the supernatants was determined using Pierce™ BCA Protein Assay Kit (Thermo Fisher Scientific, Waltham, MA, USA) for normalization of further measurements. Cyto-/chemokines were analyzed by MILLIPLEX MAP Mouse Cytokine/Chemokine Immunology Multiplex Assay (EMD Millipore, Billerica, MA, USA) and measured by Luminex 200™ (Luminex, Austin, TX, USA).

### Mathematical Model

We applied mathematical modeling to computationally simulate the tumor response as a function of MSV-nAb-PTX-coupled macrophages differentiating into M1- and M2 subtypes. As described in our previous work (25), the model (58–61) simulates viable and necrotic tissue in hepatic metastases, including the transport of macrophages and molecules through this tissue. The tumor growth is obtained through balance of cell proliferation and death. Proliferation depends on adequate oxygen and cell nutrients, while death is induced by levels of oxygen below a threshold of viability as well as drug above a certain level of cytotoxicity. Values for the model parameters were calibrated to our experimental data as in Ref. (25, 58–61). We simulated release of paclitaxel from nAb-PTX carried by nanovector-loaded macrophages infiltrating the tumor tissue and differentiating into M1- and M2 subtypes. The model and associated parameters are further described in the Supplementary Material.

### Statistical Analysis

All quantitative parameters are presented as mean values with SD. Statistical analysis was performed by *t*-test for unpaired samples using Graphpad Prism software, with *p-*value <0.05 accepted as indicative of significant difference, <0.01 as a statistically very significant difference.

## Ethics Statement

Animal studies were performed in accordance with approved protocols by Houston Methodist Research Institute Institutional Animal Care and Use Committee (IACUC) (AUP-0514-0032).

## Author Contributions

BG, FL, and HF conceived the idea and designed the research. FL and TN performed *in vitro* experiments and analyzed the data. FL and MW analyzed the live-cell imaging data. FL and KY performed *in vivo* studies. LC and HF developed computational models and therapy simulations. XL fabricated MSV. FL, HF, and BG wrote the manuscript. All the authors reviewed and approved the manuscript.

## Conflict of Interest Statement

The authors declare that the research was conducted in the absence of any commercial or financial relationships that could be construed as a potential conflict of interest.
