# Supplementary material for: Macrophage Polarization Contributes to the Anti-Tumoral Efficacy of Mesoporous Nanovectors Loaded with Albumin-Bound Paclitaxel
Source: Front Immunol. 2017 Jun 16;8:693. doi: 10.3389/fimmu.2017.00693 (PMC5472662; doi:10.3389/fimmu.2017.00693)
Supplement: Supplementary file 1 [file Presentation_1.pdf]

Supplementary information

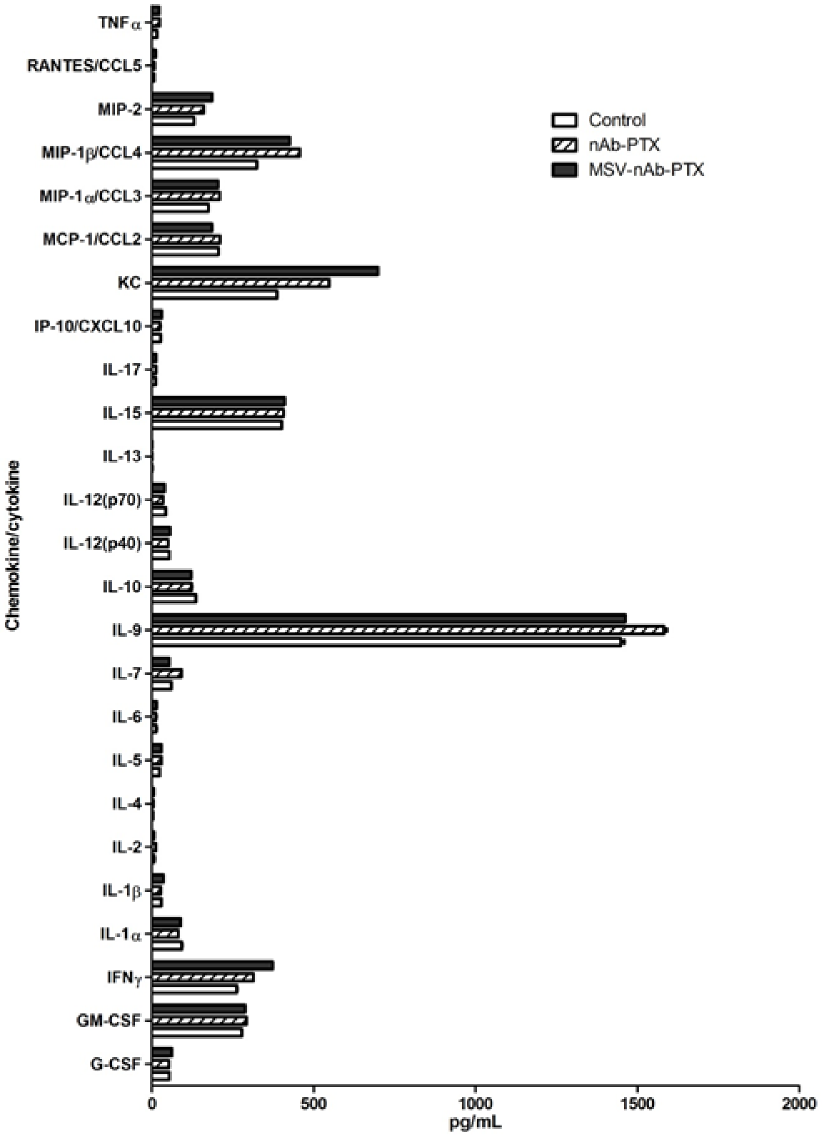

Supplementary figure 1. Macrophage cyto-/chemokine release as a function of treatments with nAb-PTX and MSV-nAb-PTX. Macrophages were cultured with treatment for 3 days and supernatants were collected for the analysis of cyto- and chemokines. Cytokine release was analyzed using MILLIPLEX MAP Mouse Cytokine/Chemokine Immunology Multiplex Assay (EMD Millipore, Billerica, MA) and measured by Luminex 200^{TM}(Luminex, Austin, TX).

Methods

Mathematical model of tumor response to MSV- nAb-PTX

We extend our model in [1] to simulate the effect of macrophage polarization into M1 and M2 subtypes on the tumor response to MSV-nAb-PTX therapy to treat breast cancer metastases to the liver. We summarize the model formulation as follows.

1. Tumor growth

The tumor model component is based on [2]. Briefly, the tumor tissue is denoted by Ω and its boundary by Σ. In general, tumor tissue may have a proliferating region Ω_P (typically in the order of 100-200 μm) in which cells have sufficient oxygen and nutrients, a hypoxic region Ω_H in which oxygen and nutrients are sufficient for survival but not for proliferation, and a necrotic region Ω_N in which oxygen and nutrients are insufficient for survival. The tumor growth velocity (non-dimensionalized) is implemented via a generalized Darcy's law [2]:

$$\mathbf{v}_c = -\mu \nabla P + \chi_E \nabla E \qquad \text{[Equation 1]}$$

where μ is cell-mobility representing the net effects of cell-cell and cell-matrix adhesion, P is oncotic pressure, χ_E is haptotaxis, and E is ECM density. Definitions for χ_E and E are in [2]. By assuming that the cell density is constant in the proliferating region, the overall tumor growth is associated with the rate of volume change:

$$\nabla \cdot \mathbf{v}_c = \lambda_p \qquad \text{[Equation 2]}$$

where λ_p is non-dimensional net proliferation rate (see below). Here, the cell density is assumed not to exceed 70% of the total tissue, with the remainder composed of extracellular matrix.

The main parameters of the model and their values are described in **Supp. Table 1**.

2. *Angiogenesis*

The angiogenesis model component simulates the model by [3] and is based on [2, 4], representing blood flow, vascular leakage and vascular network remodeling due to wall shear stress and mechanical stresses imposed by the tumor tissue. The angiogenesis model is described in detail by [2, 4]. We simulate a simplified liver vascular organization composed of square elements, acknowledging that in biological reality these elements are heterogeneously delineated by the sinusoids between the portal tracts and central veins at high density. As the tumor grows within this vascular environment, the tissue may experience heterogeneous access to elements diffusing from the vasculature, which may depend on tissue pressure as well as distance from the nearest vascular source.

3. Transport of oxygen

Oxygen σ is simulated to be transported from the location of the vasculature, supplied at rates λ_{neo}^{σ} and λ_{pre}^{σ} from the neo- and pre-existing vasculature, respectively, diffuse with a coefficient D_σ, taken up both by normal cells (with a rate $\lambda_{tissue}^{\sigma}$) and tumor cells ($\lambda_{tumor}^{\sigma}$ in the proliferating region and q_σ in the hypoxic region), and decay (with rate λ_{V}^{σ}) in the necrotic regions. The formulation is [2]:

$$0 = \nabla \cdot \left(D_\sigma \nabla \sigma \right) - \lambda^\sigma(\sigma)\sigma + \lambda_{ev}^\sigma(\mathbf{x}, t, \mathbf{1}_{vessel}, p, \sigma, h) \qquad \text{[Equation 3]}$$

$$\lambda^\sigma = \begin{cases} \lambda_{tissue}^\sigma & \text{outside } \Omega \\ \lambda_{tumor}^\sigma & \text{in } \Omega_P \\ q_\sigma(\sigma) & \text{in } \Omega_H \\ \lambda_N^\sigma & \text{in } \Omega_N \end{cases} \qquad \text{[Equation 4]}$$

where $\mathbf{x}$ is position in space, t is time, $\mathbf{1}_{vessel}$ is the characteristic function for vasculature (equals 1 at vessel locations and 0 otherwise), p is the tumor (solid) pressure, and h is the hematocrit in the vascular network related to oxygen extravasation (following [2]). The extravasation is modulated by the extravascular interstitial pressure p_i scaled by the effective pressure p_e, with k_{p_i} being the weight of the convective transport component of small molecules [5]:

$$\lambda_{ev}^\sigma = \overline{\lambda}_{ev}^\sigma \mathbf{1}_{vessel}(\mathbf{x}, t) \left(\frac{h}{\overline{H}_D} - \overline{h}_{\min} \right)^+ \left(1 - k_{p_i} \frac{p_i}{p_e} \right) (1 - \sigma) \qquad \text{[Equation 5]}$$

where $\overline{\lambda}_{ev}^\sigma$ is the constant transfer rate from both pre-existing and tumor-induced vessels. Constants $\overline{H}_D$ and $\overline{h}_{\min}$ respectively represent normal and minimum blood hematocrit required for oxygen extravasation.

4. Macrophages

Undifferentiated macrophages extravasate from the vasculature in proportion to the local concentration of macrophage chemoattractants (e.g., pro-angiogenic factors released by tumor cells), and preferentially migrate towards tissue regions (e.g., hypoxic tissue) along the increasing gradient of these chemoattractants. Macrophages undergo polarization into M1 or M2 subtypes in the vicinity of the tumor microenvironment based on the ratio of pro-M1 and pro-M2 macrophage factors being released by viable tumor cells in response to MSV-nAb-PTX. This ratio was matched to the experimental observations (**Figure 2d**). M1 subtypes are simulated to release nitric oxide, which inhibits cell viability, while M2 subtypes release tumor growth factors (e.g., TGF-β), which promote cell proliferation. The strength of these opposing effects was estimated so that their combination would lead a simulated tumor to match the growth rate of an untreated lesion, as experimentally measured in our previous work [1]. **Table 1** lists the values for the macrophage-related parameters. The rate of differentiation between the two subtypes was calibrated to achieve the average M1:M2 ratios observed experimentally (**Figure 2d**): control (1.0:1.8) and MSV-nAb-PTX (1.2:1.0) treated tumors *in vivo*. Further, M1 macrophages were simulated to penetrate deeper than the M2 subtypes into the tumor lesion to replicate this effect observed in the experiments (~2x higher number of M1 vs. M2 subtypes within 50 µm of the tumor center, **Figure 3b**). This was modeled as an additional chemoattractant selectively influencing M1 movement with increasing concentration towards the center of the lesion. Additionally, the macrophages act as point sources of drug to simulate the release of Paclitaxel from the MSV-nAb-PTX formulation.

5. Transport of drug

As in [1], the transport of drug s by macrophages is simulated at the location ($\mathbf{1}_{macrophage}$) of each macrophage:

$$0 = \nabla \cdot \left(D_s \nabla s \right) - \lambda^s(s)s + \lambda_{ev}^s(\mathbf{x}, t, \mathbf{1}_{macrophage}, p, s, h) \qquad \text{[Equation 6]}$$

The drug release is described as:

$$\lambda_{ev}^{s} = \overline{\lambda}_{ev}^{s} \mathbf{1}_{macrophage}(\mathbf{x},t)(\frac{C^{t}}{\overline{C}_{s}} - s) \quad \text{[Equation 7]}$$

where $\overline{\lambda}_{ev}^{s}$ is the constant transfer rate of drug from the macrophage. The drug level in the MSVs carried by the macrophages is initially $\overline{C}_s$; in time the release is assumed to be of the form $C^t = \overline{C}_s e^{-\alpha t}$, where the decay α is estimated experimentally [5]. This assumes first order kinetics, for which the release is concentration dependent.

The boundary conditions for all the diffusion equations are $\frac{\partial B}{\partial n} = 0$ (zero Neumann condition).

6. Effects of Macrophage Polarization

The cytotoxic effect λ_{M1} of the M1 subtypes is simulated to affect tissue proportional to the concentration λ_{NO} of nitrous oxide released in the immediate vicinity of the macrophage ($\mathbf{1}_{\boldsymbol{M1}}$), since nitrous oxide has a short half-life *in vivo* with limited diffusion distance:

$$\lambda_{M1} = \lambda_{NO} \mathbf{1}_{\boldsymbol{M1}} \quad \text{[Equation 8]}$$

M2 macrophages release diffusible growth factors (e.g. TGF-β) which promote tumor proliferation; their effect on cell proliferation is modeled as a first approximation as follows:

$$\frac{d\lambda_{M2}}{dt} = \lambda_F F(1 - (\lambda_M + \lambda_{M2})) \quad \text{[Equation 9]}$$

where λ_{M2} is the M2-induced mitosis rate, which is additive to the baseline mitosis rate λ_M of proliferating tissue, and F is the diffusible growth factor concentration (dimensionless units) released from M2 macrophages. The growth factor effect on the proliferation is λ_F. The change in time of the λ_{M2} effect on the proliferation decreases as the net proliferation $(\lambda_M + \lambda_{M2})$ approaches its maximum value of 1 day^{-1}. M2 macrophages in the model can also stimulate the quiescent (hypoxic) tumor cells to proliferate, albeit at lower rates than well perfused tissue (see below).

7. Drug effect on the tumor

Following [4], we assume that the net proliferation rate λ_P in **Equation 2** is proportional to the local level of oxygen and nutrients present. This rate is modulated by the rate of drug-induced death $\bar{\lambda}_{effect}$, while $\overline{C}_s$ rescales the drug concentration s in the tissue. Only proliferating cells are affected by the drug in order to simulate the cell-cycle dependent effect of Paclitaxel, and cell death is assumed to be an instantaneous process[5]. The formulation is as follows:

$$\lambda_p = \begin{cases} 0, & \text{outside } \Omega \\ (\lambda_M + \lambda_{M2})\sigma(1 - \bar{\lambda}_{effect} s) - (\lambda_A + \lambda_{M1}). & \text{in } \Omega_p \\ \lambda_{M2}\sigma - (\lambda_A + \lambda_{M1}), & \text{in } \Omega_H \\ -G_N, & \text{in } \Omega_N \end{cases} \quad \text{[Equation 10]}$$

where λ_A is the natural apoptosis rate and G_N is the non-dimensional rate of volume loss in the necrotic regions assuming that cellular debris is constantly degraded and fluid is removed. The M1 subtype affects both cycling (proliferating) and quiescent (hypoxic) tissue, as the cytotoxic mechanism is assumed to be cell-cycle independent.

8. Model Calibration

Following our previous work [1], the lesion growth was simulated in parallel with the dynamic drug distribution. Since the number of cancer cells is a function of lesion size, we estimate that a $1mm^3$ tumor lesion can contain up to $3x10^6$ cells [6], with about 10% of these cells being macrophages. In the simulations, we conservatively assume that the number of macrophages recruited to the lesion is ~25% of that expected *in vivo* ($2.78x10^4$ macrophages/mm^3). From the experimental data [1], we know that there are at least 10 MSV per macrophage, and that there is approximately 1.5mg of nAb-PTX per 10^9 MSV. Since 10% of nAb-PTX consists of Paclitaxel, this implies that the drug typically retained by single macrophages is 0.0015ng PTX. Given a concentration of 0.0015 ng nAb-PTX per macrophage (which represents 4.167 x 10^{-5}g nAb-PTX/mL, based on our calculation of a minimum 27,778 drug-carrying macrophages/mm^3 required to achieve an $EC5_0$ of 125ng ABX/ml equivalent to monolayer), we calibrated the tumor regression to match that observed with the 3D cell cultures [1]. This established a baseline value for the drug effect.

**Parameter**	**Value**	**Reference**
Maximum tumor proliferation rate	1 day^{-1}	Measured *in vitro*
Tumor tissue threshold for hypoxia	0.5750	Calibrated to match 3D cell culture
Tumor tissue threshold for necrosis	0.5325	Calibrated to match 3D cell culture
Oxygen diffusivity	1 (*)	[4]
Oxygen transfer rate from vasculature	5 (*)	[4]
Oxygen uptake rate by proliferating tumor cells	1.5 (*)	[4]
Oxygen uptake rate by hypoxic tumor cells	1.3 (*)	[4]
Oxygen uptake rate by tumor microenvironment	0.12 (*)	[4]
Oxygen decay rate	0.35 (*)	[4]
ABX transfer rate from MSV-nab-PTX macrophages	5 (*)	[5]
ABX diffusivity	0.25 (*)	Estimated from experimental data
ABX uptake rate by proliferating tumor cells	1.5 (*)	[5]
ABX decay rate	20 hr. half-life	[7]
MSV per MSV-nab-PTX macrophage	10	Measured *in vitro*
ABX per MSV	0.0015 ng	Measured *in vitro*
Paclitaxel per ABX molecule	10%	[7]
ABX *in vitro* EC50 (48 hrs.) for 4T1 cells (monolayer)	125 ng/mL	Measured *in vitro*
Percentage of macrophages per tumor lesion total cells	10%	Measured *in vitro*
Resistance differential between monolayer and 3D cell culture when macrophages are present	1	Measured *in vitro*
Number of macrophages needed *in vivo* to attain EC50 *in vitro*	27,778 / mm^3	Calculated from experimental data
M1-induced death rate	5 / s	Estimated from experimental data
M2 growth factor strength	1000 /s	Estimated from experimental data
ABX-induced death effect	8400	Calibrated to match 3D cell culture

Supplementary Table 1. Main parameters of the computational model and their associated values. (*) Value is rescaled by the square of the simulation system characteristic length (1 cm) and divided by the system characteristic time (1 sec) multiplied by the oxygen diffusivity [8] (1 x 10^{-5} cm^2 s^{-1}).

References

1. F. Leonard, L. T. Curtis, P. Yesantharao, T. Tanei, J. F. Alexander, M. Wu, J. Lowengrub, X. Liu, M. Ferrari, K. Yokoi, H. B. Frieboes and B. Godin, *Nanoscale*, 2016, **8**, 12544-12552.
2. P. Macklin, S. McDougall, A. R. A. Anderson, M. A. J. Chaplain, V. Cristini and J. Lowengrub, *Journal of Mathematical Biology*, 2009, **58**, 765-798.
3. S. R. McDougall, A. R. A. Anderson and M. A. J. Chaplain, *J Theor Biol*, 2006, **241**, 564-589.
4. M. Wu, H. B. Frieboes, S. R. McDougall, M. A. J. Chaplain, V. Cristini and J. Lowengrub, *J Theor Biol*, 2013, **320**, 131-151.
5. A. L. van de Ven, M. Wu, J. Lowengrub, S. R. McDougall, M. A. J. Chaplain, V. Cristini, M. Ferrari and H. B. Frieboes, *AIP Advances*, 2012, **2**.
6. L. Spinney, *Nature*, 2006, **442**, 736-738.
7. *Abraxane® (paclitaxel protein-bound particles for injectable suspension)(albumin-bound)*, Celgene Inc. , 2015.
8. L. J. Nugent and R. K. Jain, *Cancer Research*, 1984, **44**, 238-244.
